# Conference Report: WORKSHOP ON CHARACTERIZING DIAMOND FILMS Gaithersburg, MD February 27–28, 1992

**DOI:** 10.6028/jres.097.017

**Published:** 1992

**Authors:** Albert Feldman, Charles P. Beetz, Michael Drory, Sandor Holly

**Affiliations:** National Institute of Standards and Technology, Gaithersburg, MD 20899; Advanced Technology Materials, 7 Commerce Dr., Danbury, CT 06810; Crystallume, 125 Constitution Dr., Menlo Park, CA 94025; Rockwell International, 6633 Canoga Ave., Canoga Park, CA 91304

## 1. Introduction

A workshop was held at NIST on February 27–28, 1992 to discuss specific topics deemed important to the characterization of diamond films made by chemical vapor deposition (CVD) and to address the need for standards in diamond technology. The workshop was held as a follow-up to a recommendation in a recent report assessing diamond technology in Japan [1]. The audience targeted for this workshop was the producers and potential users of CVD diamond technology in the United States. University scientists were invited as experts in properties measurements. There were 35 participants in the workshop.

The workshop consisted of two components, technical presentations by active researchers on topics of the most immediate relevance to commercial applications and presentations by companies describing their needs for standards.

Rather than choose a wide variety of topics for discussion, we focussed on two technical topics, the measurement of thermal conductivity or thermal diffusivity for heat dissipation applications, and the measurement of mechanical properties of diamond for cutting tool applications. These properties are important for other applications as well. The topics were selected on the basis of responses to a questionnaire sent to potential participants in the workshop. Presentations describing the NIST Advanced Technology Program and NIST facilities that might be useful to diamond researchers were also given.

After the workshop, an evaluation form was sent to all registrants. The purpose of the form was to determine the usefulness of the workshop, the desire for further workshops, and the need for standards related activities. The results of this questionnaire will be included in a NIST Internal Report to be distributed to workshop participants.

The sections that follow contain summaries of the three main sessions of the workshop. The principal conclusions to be drawn from the workshop include the following:

- There is a need to standardize characterization methods so that experimental data, measured at different sites by different workers, may be meaningfully compared.
- There is a need for specimens with well characterized properties. The first priority identified was a need for specimens to measure thermal conductivity or thermal diffusivity in interlaboratory round-robin comparisons. Development of a Raman standard reference material would also be useful for assessing diamond quality.
- Standards for mechanical measurements would be premature at present. Only a limited number of measurements of specific properties, such as fracture toughness and hardness, have been made. Because of the great hardness of diamond, mechanical measurements are difficult to make and difficult to interpret.
- Other workshops should be held at intervals of about 1 year, focusing on other topics, such as optical and electrical properties.
- Working groups dealing with standards for specific topical areas should be established, possibly under the auspices of a standards organization such as ASTM.
- NIST has an important role to play in coordinating and facilitating the development of standards for diamond technology and in developing standard reference materials.

## 2. Thermal Properties Measurement

The session on thermal properties consisted of eight speakers presenting different measurement techniques.

The first paper was given by John Graebner from AT&T Bell Laboratories. He gave a brief review of the physical mechanisms responsible for thermal conductivity in diamond and then discussed measurements of thermal conductivity he has made on natural single crystal diamonds and on CVD diamond films. Results of thermal conductivity measurements obtained with three different techniques were presented. Dr. Graebner showed that the thermal conductivity varied through the thickness dimension of a CVD diamond film; the bottom surface, the surface where film growth began, had one fourth the thermal conductivity of the top surface. Moreover, the thermal conductivity was anisotropic; the thermal conductivity for heat flow parallel to the specimen surfaces was lower than the thermal conductivity for heat flow perpendicular to the specimen surfaces. In a film 300 μm thick the thermal conductivity near the top surface was found to be 22 W·cm^−1^·K^−1^, which is comparable to the value for high quality single crystal diamonds. By modeling the dependence of the thermal conductivity on grain size, he predicted thermal conductivity of 25–28W·cm^−1^·K^−1^ for 1 mm sized grains, an interesting result because these values are unexpectedly large.

The second speaker was Jan Vandersande from the Jet Propulsion Laboratory, who discussed the temperature dependence of the thermal conductivity of white natural diamonds. Thermal diffusivity measurements were made from room temperature to 1000 K by means of a flash lamp measurement technique. The room temperature thermal conductivity was 23W·cm^−1^·K^−1^ and at 1200 °C was 5 W·cm^−1^·K^−1^. This technique can also be used to measure the specific heat. As an aside. Dr. Vandersande demonstrated that due to instrumentation limitations, the true electrical resistivity of diamond can be much larger than measurements would indicate.

David Cahill from the University of Illinois discussed measuring thermal conductivity by an ac measurement technique, called the 3-omega method. In this method, a thin metal wire is deposited onto the diamond surface. The wire with electrical resistance, *R*, acts both as the heat source and as the thermometer. An alternating current, *I*, produces resistive heating, *I^2^R;* the resultant temperature rise in the specimen produces a change in resistance of the wire. This change in resistance is a measure of the temperature. The technique is called the 3-omega method because the measurements are made at the third harmonic of the ac current frequency. This technique is immune to the effects of thermal radiation and is well suited for measurements both on bulk specimens or on thin films. Dr. Cahill measured the thermal conductivity of a range of carbon based materials, including C_60_; the values spanned four orders of magnitude.

Ronald Tye, a consultant to Sinku-Riko, discussed a commercially available instrument that uses a modified ac calorimetry method based on optical heating to measure both the thermal diffusivity and the heat capacity of thin films. Dr. Tye showed results for diamond films and for standard samples of nickel, silicon, alumina, and stainless steel.

Donald Morelli of the General Motors Research Laboratories showed how thermal conductivity measurements can be used to study defects in diamond. He reviewed the sources of error in the classic steady state method of measuring thermal conductivity. Results of thermal conductivity measurements conducted over a range of temperatures down to cryogenic temperatures on films deposited by plasma assisted CVD and hot filament CVD were reported. Although the films prepared by the two methods were alike in almost all respects, the films prepared by the hot filament method showed an anomalous behavior at low temperature that has not yet been explained.

P. K. Kuo of Wayne State University discussed the measurement of the thermal diffusivity of diamond by the method of photothermal deflection. In this technique, a probe laser beam skimming the surface of the specimen is deflected by refractive index gradients that occur in the air above the specimen due to heating from a thermal wave propagating in the specimen. Results were reported for diamond films down to 5 μm thick and for synthetic single crystal diamonds about 3.5 mm thick. Values of thermal diffusivities ranged from less than 1 to 9.2 cm^2^·s^−1^ Dr. Kuo also discussed a modification to the technique in which the probe beam passes directly through the crystal under measurement.

Grant Lu from the Norton Company discussed the requirements for a measurement technique that could be used effectively as a process monitor or a quality control monitor. First he described the converging thermal wave technique that he uses to measure thermal diffusivity. Then he discussed the results of a thermal conductivity round-robin, in which diamond samples taken from a contiguous section of a 10 cm diameter diamond plate were sent to several laboratories for thermal conductivity measurements. Each laboratory used a different method of measurement. The values of thermal conductivity displayed considerable scatter, generally ranging from 12.3 to 16.7 W·cm^−1^·K^−1^ with one result greater than 20 W·cm^−1^·K^−1^. The cause of the scatter in the data was discussed but not resolved.

A. Feldman from NIST discussed the use of photothermal radiometry for measuring the thermal diffusivity of diamond. Two different geometries for making the measurement were discussed, one using a laser heating beam with a cylindrically symmetric Gaussian profile and another using a laser heating beam with a cylindrically symmetric ring profile which results from placing an axicon lens into the beam. The results of numerical simulations for the phase and magnitude of the surface temperature as a function of the chopping frequency of the laser were presented. He also discussed the importance of knowing the laser beam characteristics and the importance of having the sample precisely focussed on the detector.

## 3. Mechanical Properties

Characterizing the mechanical properties of CVD diamond requires that several broad topical areas be explored. These include elastic properties, plastic properties, and fracture properties.

In the case of a composite system, such as diamond/silicon, interface properties are an additional consideration. Examples of elastic properties to be examined include Young’s modulus and Poisson’s ratio. The hardness is a primary measure of plastic behavior, while fracture toughness characterizes the fracture behavior of a material. In this workshop, each broad area of mechanical behavior was briefly considered for the purpose of assessing the status of testing methods and practices in preparation for standards. As described below, several areas of mechanical characterization of CVD diamond are generally unexplored, requiring considerably more attention in coming years.

Of the mechanical properties, the elastic behavior of CVD diamond has received the most attention by researchers because of the potential benefits diamond brings to window applications. Recent work on the elastic properties of CVD diamond was presented by C.A. Klein of Raytheon in which attention was directed toward the elastic constants of polycrystalline diamond derived from single crystal values. Dr. Klein emphasized the considerable range in measured values of Young’s modulus obtained by different researchers. He pointed out the need for careful sample description and careful analysis of the test method. In addition, he pointed out that the Poisson’s ratio should be 0.07 rather than 0.2, the latter value being often cited in the literature.

Considerable work has been done on the plastic behavior of CVD diamond. Presentations by C. V. Cooper of United Technologies and C. J. McHargue of the University of Tennessee discussed the use of nanoindentation to obtain the hardness of CVD diamond. The dependence of hardness on process variables, such as the amount of methane in the deposition feed gas, were discussed. Dr. Cooper discussed the experimental difficulties caused by surface roughness. Dr. McHargue discussed the experimental difficulties associated with the choice of indenter geometry. Dr. McHargue also pointed out the necessity of relating a particular test procedure to a specific definition of hardness.

The fracture behavior of CVD diamond was discussed in several of the presentations. J. J. Mecholsky of the University of Florida and P. Klocek of Texas Instruments discussed the measurement of the *film* fracture toughness by means of a surface analysis technique, while M. D. Drory discussed an indentation method. Professor Mecholsky’s method showed considerable potential for determining the fracture toughness for a variety of specimen geometries, without the need for special specimen preparation.

M. D. Drory discussed quantifying adhesion through the measurement of *interface* fracture toughness. In particular, a first-order analysis was presented for determining interface toughness from a brale indentation measurement. The test was applied to diamond on relatively ductile substrates, such as cemented carbide. Considerably more work is needed to develop adhesion tests suitable to diamond.

## 4. The Need for Standards

There are several good reasons to establish standards for the evolving field of CVD diamond film technology. One of the purposes of this workshop was to identify the needs for standards in industry. Representatives of six companies agreed to discuss their views on this subject. The speakers were J. W. Mitchell of AT&T Bell Laboratories, M. D. Drory of Crystallume, K. Gray of Norton Diamond Films, S. Holly of Rockwell International, P. Klocek of Texas Instruments, and C. J. Robinson of the Raytheon Company. All of the companies are currently conducting CVD diamond film research. While some of the companies are commercial producers of the material, others are users of CVD diamond and are applications oriented.

The presentations described the current methods of characterizing diamond films used by these companies and the importance of the measurements to quality assurance of the CVD diamond material as related to a variety of company specific applications. The most important application areas discussed were thermal (heat sinks), optical (infrared windows and domes), tribological, and electronic.

All of the presenters agreed on the value of standards to meet performance criteria in different applications. There is a need to standardize the characterization methods so that experimental data, measured at different sites by different workers, may be meaningfully compared. To that end, a discussion began on the value of round-robin type tests, where the same sample is measured by different laboratories, often using different measuring methods. The importance of thorough documentation of the methods used in these measurements was emphasized. In fact, the question of what “thorough documentation” means directly emphasized the need for standards, the focus of this workshop.

It became obvious during these discussions that different applications require different types of measurements. There was also a general consensus that measurements procedures bridging different disciplines are needed. Because of the interdisciplinary requirements of the technology, the activities of workers with different areas of experise will require coordination. These workshops provide such a forum.

It may be practical or it may even be necessary to form specialty groups to formulate standards in separate application areas. Development of standards would start with the definition of simple concepts, such as the meaning of “high quality diamond” and the interpretation of a particular characterization, such as a Raman spectrum or a thermal conductivity measurement.

In order for the industrial community to derive immediate benefits from these standardization activities it will be important to promote exchange of information through meetings and publications. It was also proposed that a data base of CVD diamond material properties, based on accepted “standardized” methods, be established. The data base would require updating on a continual basis.

There was general agreement during the discussions in this session, that focussing on just one characteristic at a time was most valuable. The previous day’s session on thermal properties, which contained technical papers describing methods of measuring thermal conductivity of CVD diamond, was an excellent example of the value and necessity of focusing on a single characteristic. Agreement was reached among many workshop participants, that future workshops on standardization should highlight other characteristics, such as optical or electrical properties.

A pertinent final presentation in this session was made by E. S. Etz of NIST who discussed development of a Standard Reference Material for Raman spectroscopy of CVD diamond [2]. It was generally agreed that such a standard would be useful. Questions to be considered are: how many specimens would be needed, how should the specimens be obtained, what specimen dimensions are needed, and how extensively should the specimens be characterized by other methods?

## References

[1] Feldman A, Schwartz LH U.S. Assessment of the New Diamond Technology in Japan. Natl Inst Stand Technol.

[2] Etz ES, Tzeng Y, Yoshikawa M, Murakawa M, Feldman A (1991). Applications of Diamond Films and Related Materials.

